# Nanoscale Topographical Characterization of Orbital Implant Materials

**DOI:** 10.3390/ma11050660

**Published:** 2018-04-24

**Authors:** Marco Salerno, Andrea Pietro Reverberi, Francesco Baino

**Affiliations:** 1 Materials Characterization Facility, Istituto Italiano di Tecnologia (IIT), Via Morego 30, 16163 Genova, Italy; marco.salerno@iit.it; 2 Department of Chemistry and Industrial Chemistry, Università di Genova, Via Dodecaneso 31, 16146 Genova, Italy; reverb@dichep.unige.it; 3 Institute of Materials Physics and Engineering, Applied Science and Technology Department, Politecnico di Torino, Corso Duca degli Abruzzi 24, 10129 Torino, Italy

**Keywords:** bioceramic, glass-ceramic, orbital implant, roughness, atomic force microscopy, ocular surgery, enucleation

## Abstract

The search for an ideal orbital implant is still ongoing in the field of ocular biomaterials. Major limitations of currently-available porous implants include the high cost along with a non-negligible risk of exposure and postoperative infection due to conjunctival abrasion. In the effort to develop better alternatives to the existing devices, two types of new glass-ceramic porous implants were fabricated by sponge replication, which is a relatively inexpensive method. Then, they were characterized by direct three-dimensional (3D) contact probe mapping in real space by means of atomic force microscopy in order to assess their surface micro- and nano-features, which were quantitatively compared to those of the most commonly-used orbital implants. These silicate glass-ceramic materials exhibit a surface roughness in the range of a few hundred nanometers (S_q_ within 500–700 nm) and topographical features comparable to those of clinically-used “gold-standard” alumina and polyethylene porous orbital implants. However, it was noted that both experimental and commercial non-porous implants were significantly smoother than all the porous ones. The results achieved in this work reveal that these porous glass-ceramic materials show promise for the intended application and encourage further investigation of their clinical suitability.

## 1. Introduction

The morphological properties of biomedical implant surfaces (e.g., texture, roughness) are known to greatly influence the cell and tissue responses in vitro and in vivo [1,2,3,4]. Early studies carried out in the 1990s on metallic prosthetic implants provided the first evidence that osteoblastic cells preferably attach and spread on titanium surfaces exhibiting a diffused micro-scale roughness [2,5,6]. Over the last two decades, advanced investigations at the frontier between (bio)materials science, biology, and medicine have allowed scientists to better elucidate the role played by micro- and nano-topography of the implant on cell–biomaterial interactions [5]. It was generally observed that the micrometric and nanometric peaks and valleys of the implant surface can affect the organization of cell cytoskeleton, and hence the intracellular transduction signaling pathways [7,8]. 

However, there are some biomedical applications for which the presence of a surface micro- or nano-roughness may not be a goal and should be minimized (e.g., cardiovascular devices (heart valves, coronary stents) [9] and orbital implants [10]). Orbital implants substitute a diseased ocular globe after its surgical removal through enucleation due to either cancer (e.g., retinoblastoma in children), extensive orbito-facial trauma, or ophthalmic infections irresponsive to pharmacological therapy [11]. Over the years, orbital implant design evolved from non-porous balls made of glass, silicone, or poly(methyl methacrylate) (PMMA) to macroporous spheres (hydroxyapatite, polyethylene, alumina) that permit better biointegration [11]. 

**Figure 1:**
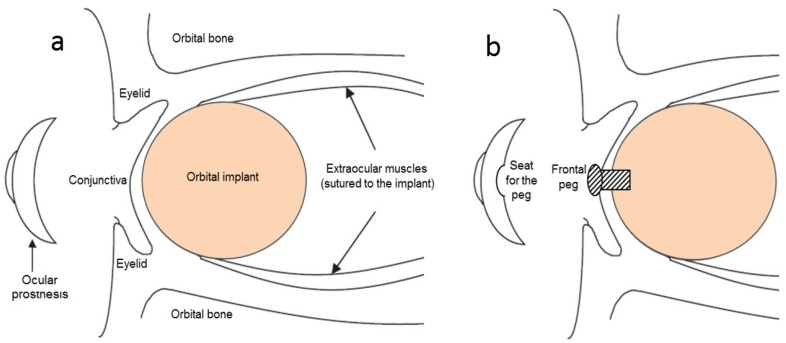
Scheme showing the placement of a spherical orbital implant following enucleation surgery. The connection between the orbital implant and the aesthetic ocular prosthesis can be (**a**) indirect, if the conjunctiva completely coats the frontal part of the implant, or (**b**) direct, by the use of a peg.

Most orbital implants are “buried” under the patient’s conjunctiva to isolate the implant from the external environment, thus minimizing the risk of postoperative bacterial colonization and infections (see Figure 1a). Proper aesthetics is achieved by making use of an ocular prosthesis, which is an acrylic insert—similar to a large and thick contact lens—that is sandwiched between the eyelids and the conjunctiva [12]. The ocular prosthesis is painted to closely match the appearance of the living contralateral eye, and it can be temporarily removed for cleaning and when the patient goes to sleep. If the extraocular muscles are attached to the orbital implant, some movements may be transmitted to the overlying ocular prosthesis, which exhibits a life-like motility.

Currently, there is convincing evidence that porous orbital implants lead to better performances and clinical outcomes as compared to non-porous balls [13]. Porous implants are typically characterized by a three-dimensional (3D) network of large (100–500 µm) and interconnected macropores which allow the ingrowth of vascularized connective tissue. Fibrovascularization, which typically occurs from 4 to 6 weeks postoperatively, offers several key advantages [14], including (i) better anchorage of the implant to host soft tissues and minimal risk of extrusion; (ii) reduction of the risk of implant infection due to the good blood supply and immune surveillance within the porous material; and (iii) possibility of implant pegging (i.e., the establishment of a direct connection between implant and aesthetic ocular prosthesis by a small titanium peg in order to further improve the motility—Figure 1b). 

However, there still are some drawbacks to porous orbital implants, especially if they are made of ceramic materials. The major drawbacks are the high cost and the non-negligible risk of exposure due to the irregular surface of the implant. The latter problem is associated to the presence of stiff micrometric crystals that can protrude from the implant struts and erode the overlying conjunctiva in combination with the repetitive movements of the implant governed by the extraocular muscles [15,16].

In previous works, we produced porous silicate glass-ceramics by sponge replication for possible use as orbital implant materials [17] and analyzed their surface by scanning electron microscopy (SEM) and stylus profilometry [18]. Interestingly, these early investigations suggested that the surface roughness of these novel implants was significantly lower than that of commercially-available ceramic implants, thus strongly motivating further research. The present study aims at expanding those promising results and reports the advanced characterization of orbital implants by atomic force microscopy (AFM) with a particular focus on the nanoscale roughness present on the surface of the struts. AFM was already used for the investigation of ocular biomaterials, such as polymeric intraocular lenses [19] and some commercial orbital implants [20]. Compared to SEM, AFM allows the fine structure (“ultrastructure”) of orbital implants to be characterized by measuring the surface topography, and can provide quantitative data on the implant surface roughness at both micro- and nano-scale, which are key determinants for predicting their actual suitability for clinical use.

## 2. Materials and Methods

### 2.1. Ocular Implant Materials

Experimental porous implant materials were manufactured by using two non-commercial SiO_2_-based biocompatible glasses as starting materials. These glasses, referred to as type A (57SiO_2_-34CaO-6Na_2_O-3Al_2_O_3_ mol %) and type B (45SiO_2_-3P_2_O_5_-26CaO-7MgO-15Na_2_O-4K_2_O mol %), were prepared by melting homogenous mixes of high-purity reagents (oxides, carbonates, and other appropriate salts, all purchased from Sigma-Aldrich, St. Louis, MO, USA) in a platinum crucible at 1550 °C in air for 0.5 h. The melt was cast in deionized water to obtain a “frit”, which was suitable for the further crushing process by ball-milling (Pulverisette 0, Fritsch, Idar-Oberstein, Germany). Glass powders were then sieved through stainless steel sieves (Giuliani Technologies, Torino, Italy) to obtain particles with size below 32 µm.

Porous implants derived from glasses A and B were fabricated by sponge replication as described in a previous work [18]. Briefly, 10-mm cuboids of an open-cell 45-ppi polyurethane foam were dipped into an aqueous suspension comprising glass powder (40 wt %), deionized water (54 wt %), and poly(vinyl alcohol) (6 wt %) acting as a binder. Then, the glass-coated polymeric blocks were extracted from the slurry and compressed along the xyz directions in order to squeeze the suspension out of the pores and leave a thin layer of glass powder on the sponge struts. After undergoing this impregnation–squeezing cycle three times, the samples were dried overnight at room temperature in air and then heat-treated at 950 °C for 3 h to remove the sacrificial polymer and to sinter the glass particles, thus obtaining a 3D replica of the porous template. The resulting sintered cuboids were made of biocompatible glass-ceramic materials, referred to as GCA and GCB, and exhibited adequate macropore characteristics for use as orbital implants, as previously discussed by Baino et al. [18] and summarized in Table 1. 

Three types of commercial implants were also selected for comparative purposes. These reference implants were porous balls of alumina (Bioceramic implant, FCI, Paris, France) and polyethylene (PE) (Medpor^®^, Porex Surgical, Newman, GA, USA), along with non-porous balls of PMMA and silicone (FCI, Paris, France). A description of these materials with the expected known properties is also included in Table 1.

### 2.2. Characterization

Topographical analysis was carried out for all the considered materials listed in Table 1 by means of an MFP-3D AFM (Asylum Research, Goleta, CA, USA). The AFM was operated in tapping mode with silicon probes NCHR (Nanosensors, Neuchâtel, Switzerland), having nominal cantilever resonance frequency of ~330 kHz and tip apex diameter of ~20 nm. We acquired images of 40 µm × 40 µm scan areas, with 256 × 256 pixels, with pixel-limited resolution of ~150 nm in both in-plane directions. The vertical resolution is estimated to be ~1 nm.

Because the ball-shaped samples were thicker than the maximum sample height allowed, a recessed base holder had to be used in order to let them seat under the AFM head. This holder consisted of an annular ring letting a significant portion of the ball sit under the horizontal level of the head base plate. The ball was fixed to the annular ring by placing underneath either plasticine or an elastic drum-like membrane that was cut off single-use lab gloves. 

For each 3D image of surface morphology, the following quantities were extracted from the respective distributions of heights: root mean square S_q_, which is the same as the standard deviation after subtraction of the best-fitting plane; arithmetic mean deviation S_a_; skewness S_k_, which is the third moment of the distribution; and kurtosis K_u_, which is the fourth moment of the distribution. Details of the mathematical definitions may be found in the relevant ISO standard [21]. 

### 2.3. Statistical Analysis

For each sample, at least four images at different surface positions were acquired (N ≥ 4), and the resulting quantities described above (S_q_, S_a_, S_k_, K_u_) were averaged in order to extract the mean and the standard deviation. The analysis of variance (ANOVA) was also carried out on these four parameters to assess the statistical significance of the apparent differences among the means, by means of the plotting and analysis software OriginPro 2016 (Originlab, Northampton, MA, USA). We probed the statistical significance levels α of 0.05 and 0.01, and compared all the pairs of implant materials with Tukey test.

## 3. Results and Discussion

**Figure 2:**
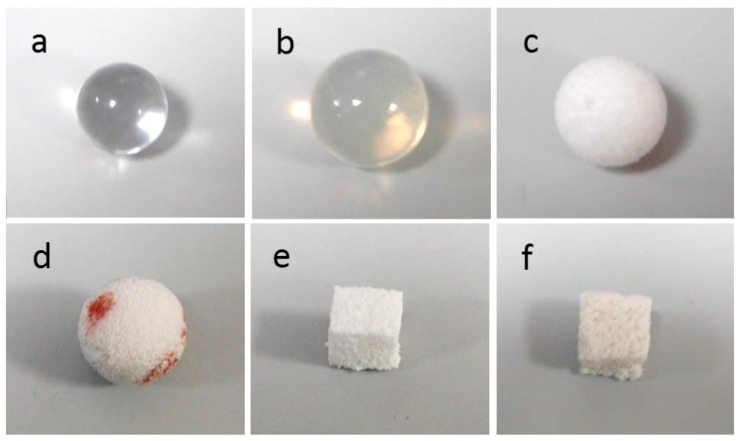
Pictures of the samples analyzed in this work: (**a**) non-porous PMMA; (**b**) non-porous silicone; (**c**) porous PE; (**d**) porous alumina (red pen marks were done as reference positions to make it easier to find the struts and land onto them with the atomic force microscope (AFM) probe tip, instead of sinking into the macropores); (**e**) porous GCA; (**f**) porous GCB. Implant dimensions are summarized in Table 1.

**Figure 3:**
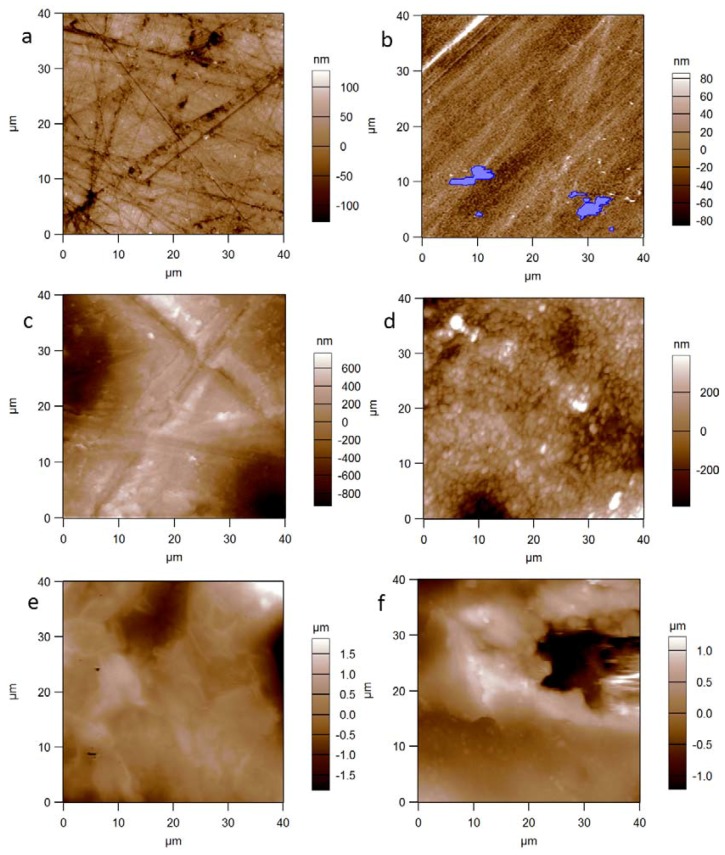
Representative topographic images of the materials’ surfaces: (**a**) PMMA; (**b**) silicone; (**c**) PE; (**d**) alumina; (**e**) GCA; (**f**) GCB.

Figure 2 displays some camera pictures of the samples investigated in this work (see also Table 1 for details). Figure 3 collects representative surface topography images of the six different samples, which are presented as an example of the observed surface morphologies. All the samples were successfully imaged by AFM, despite the occasional occurrence of some local defects such as partial line strikes (Figure 3c,d) and apparent contaminant particles overlaying the sample surface (Figure 3b), which were removed by the scan area considered for quantitative analysis by means of image masking. The sample that gave most problems during imaging was the alumina implant (Figure 2g), for which even the trabecular areas between adjacent macropores appeared to be quite rough and tilted at angles that hardly allowed the z-range of the AFM piezo-actuator (~12 µm) to cover the portion of surface imaged without saturating. However, after several trials and by rejecting the unsuccessful scans, it was possible to reach the minimum target number (N = 4) of useful images with acceptable quality. It should be noticed that the commercial macroporous alumina investigated here, probably produced by foaming method, had much larger pore size compared to the porous alumina resulting from aluminum anodization [22,23], also used in biological applications mainly for in vitro for cell cultures and successfully measured under AFM [24] thanks to the nanoscale size of its pores. 

It appears that both PMMA (Figure 3a) and silicone (Figure 3b) surfaces were flat and rather smooth (z-range of a few hundred nanometers), with typical surface lines, straight across the considered length scale. The linear features were more marked—appearing as depressed scratches—and more randomly oriented in the case of PMMA, whereas for the silicone they appeared milder and more aligned along a single direction, which could be the lay resulting from the original machining rather than a finishing step carried out to decrease the roughness. In particular for silicone, most inspected areas presented small overlying contaminated regions that could not be removed by gently cleansing with tissue and ethanol, and were thus removed from the analyzed image areas by means of masking (see blue particles in Figure 3b). 

Different from PMMA and silicone, the other four materials were inherently porous. In these cases, we carefully landed with the AFM probe tip on the top of the macropore walls, trying to avoid falling into them, and thus characterizing the roughness of the trabecular structure. In particular, the PE (Figure 3c) exhibited a straight-lines pattern similar to the former non-porous polymers and especially close to the randomly scratched surfaces of PMMA. These scratches were not apparent for the other, non-polymeric macroporous materials (Figure 3d–f). The trabecular walls of the alumina were the most difficult to track with the AFM, due to the much different mean height of the adjacent struts surrounding the macropores. Nevertheless, the single trabecular walls were locally quite smooth (Figure 3d), exhibiting rounded grain appearance. The two experimental glass-ceramics, GCA (Figure 3e) and GCB (Figure 3f), appeared qualitatively quite similar to each other in terms of morphology alone, exhibiting flat regions with intercalated middle-sized pores (diameter from a few micrometers to tens of micrometers), more marked in the case of GCB.

**Figure 4:**
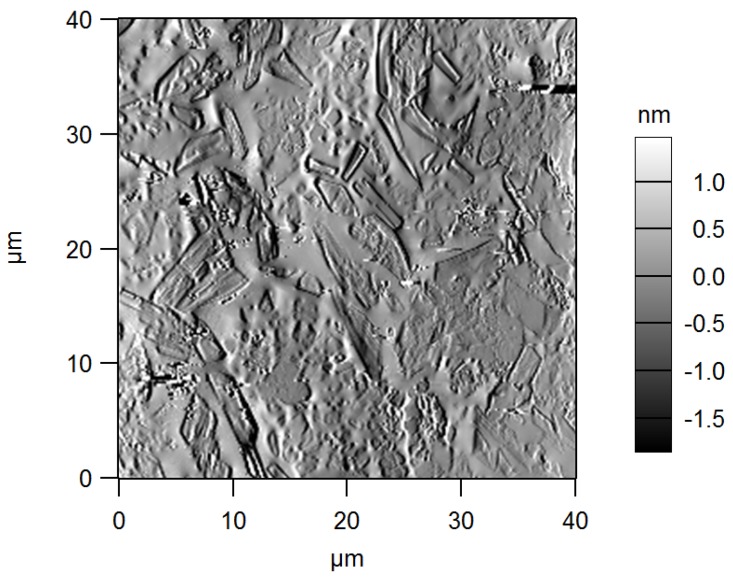
Image of additional data channel taken by AFM on GCA, simultaneously with the height one in Figure 3e: the scale reports the mean deviation in oscillation amplitude between trace and retrace scan.

The AFM also allowed us to visualize the glass-ceramic nature of GCA thanks to the images of cantilever oscillation amplitude, which is the error signal to be cancelled out in the AFM feedback circuit. This signal is sensitive to tiny local deviations in morphology, and in fact it can capture fine details, with a resulting rendering similar to that of a grazing angle topographic image. As an example, Figure 4 shows an image of mean amplitude—that is, (trace scan amplitude + retrace scan amplitude)/2—acquired simultaneously with the height image of Figure 3e. Clearly, the edges of regular polygonal wollastonite crystals embedded in an amorphous matrix of residual glass were detected, in agreement with X-ray diffraction analysis and SEM investigation reported in a previous work [17]. 

**Figure 5:**
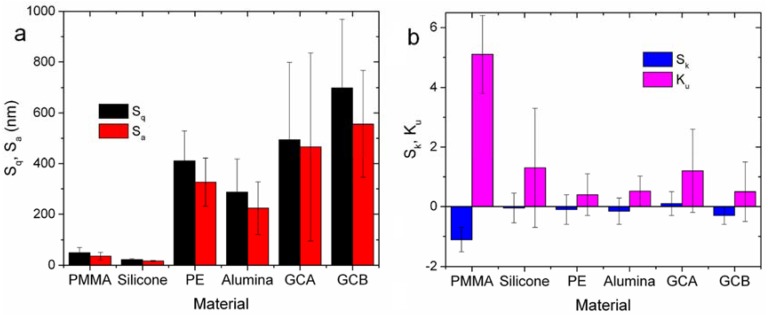
Plot of the topographical parameters of interest, as extracted from images of the surfaces similar to those examples shown in Figure 3: (**a**) roughness parameters S_q_ and S_a_; (**b**) height distribution shape parameters, S_k_ and K_u_.

The most important information that can be obtained from the 3D images shown in Figure 3 concerns the amplitude parameter S_q_, which is the root mean square of the distribution of heights and is a measurement of the surface roughness. Additionally, the higher moments of the height distributions were also calculated, namely the skewness S_k_ and the kurtosis K_u_. These describe the symmetry and the curvature of the height distributions, respectively. The sign of skewness is associated with the dominant type of features—“valleys” (negative values) or “mountains” (positive values). The kurtosis is associated with the shape of these features, which are either “spiky” or “bumpy” for values of kurtosis above or below three, respectively. The results of the mentioned analysis are listed in Table 2 and graphically shown in Figure 5.

When looking at S_q_ and S_a_ in Figure 5a, it appears that indeed PMMA and silicone had similar roughness and were both much smoother than the other—all porous—materials. In particular, for S_q_ the ANOVA indicates that the observed differences between all the pairs across the two groups—namely PMMA and silicone on the one side, and PE, GCA and GCB on the other side—were all statistically significant at the 0.01 significance level. On the contrary, only one inter-group difference was statistically significant for the GCB-alumina pair, at the mildest 0.05 level. In Figure 5a, the S_a_ is also shown, which is sometimes used to describe the roughness in the industrial context in place of S_q_. While staying on systematically lower values than the S_q_, the S_a_ ranks similar to that and the ANOVA also gives similar results. 

Fabrication (and then implantation) of orbital implants which are as smooth as possible is key for clinical success: in fact, the higher the surface roughness of the implant, the higher the risk of postoperative failure due to conjunctival abrasion as the implant moves under the action of extraocular muscles. The data collected in the present work give evidence that the roughness (S_q_ and S_a_) of commercial non-porous polymeric implants is one order of magnitude lower than that of the porous ones (Table 2). This is in apparent contradiction with most clinical literature showing that porous orbital implants—although having a higher surface roughness—are associated with a lower rate of complications (especially extrusion) compared to non-porous devices [25,26,27]. This issue can be explained considering that porous implants allow fibrovascularization, which permits small exposures to heal spontaneously and make the implant more amenable of “salvage procedures” [10]. The surface roughness of porous PE and alumina implants are statistically comparable, although the struts of the ceramic implant are smoother; this trend is in agreement with a study by Choi et al. [20]. Furthermore, these topographical data are consistent with the clinical results reported by Jordan et al. [28], who compared the success rate of the PE and alumina implants in a rabbit model. Apart from the more favorable topographical surface features, the better outcomes of alumina implants are also due to the faster rate of fibrovascularization, which is promoted by the material surface chemistry. In fact, cell and tissue adhesion are favored by hydrophilic surfaces like those of ceramics, and discouraged by hydrophobic polymers that tend to be encapsulated in a fibrous collagenous capsule [29]. From the viewpoint of topography, GCA shows promise as its surface roughness is comparable to that of porous PE and alumina. 

We now turn to the considerations about skewness and kurtosis. It should be reminded here that the use of these surface height parameters, in addition to more standard roughness parameters [30], has already demonstrated to provide useful insights for a number of different materials, such as dental restorative resins [31,32] and composite coatings [33]. In this work, due to the broad scattering between different samples’ areas, no difference appears to be statistically significant. Nevertheless, when looking at the ranking of the means, one can observe that PMMA had the most negative mean for S_k_, suggesting that the dominating features were the valley-type scratches, which are also present in alumina, GCB, and PE implants, but to a lesser extent. At the same time, K_u_ was less than three for all samples except for PMMA (again), meaning that those dominating scratches are quite sharp in shape. Overall, silicone thus emerges as the smoothest material in the broadest sense, more than PMMA even if similar when limiting the analysis to S_q_ only. From the viewpoint of the skewness, GCB shows also promise: in fact, exposing a predominance of valleys instead of peaks is expected to reduce the risk of conjunctival abrasion as the orbital implant moves.

In summary, the surface topographies of both GCA and GCB are attractive for potential applications in the field of orbital implants. Furthermore, especially if compared to alumina—which is currently considered the “gold standard” implant in enucleation—GCA and GCB are highly appealing from a technological viewpoint as well, as their fabrication based on viscous flow sintering requires lower temperatures and shorter times than that needed for making alumina products, thus allowing processing simplification and potential reduction of implant cost. The preliminary results reported in this study motivate further work on the development of implantable glasses and glass-ceramics for ocular surgery, which is an emerging and highly-challenging field of research [34,35,36,37]. Of course, while there is abundant literature demonstrating the biocompatibility of alumina, PE, silicone, and PMMA in contact with ocular and orbital tissues [38,39,40], these aspects for GCA and GCB materials remain to be assessed. However, some considerations can be made on the basis of previous results achieved on these materials for other biomedical applications. GCB was initially proposed for bone tissue engineering and exhibited an excellent biocompatibility with osteoblast-like MG-63 cells, which were shown to favorably adhere, spread, and grow on it [41,42]. GCA has not yet been tested with cells, but it was found to exhibit an inert-like behavior upon soaking in simulated body fluid [17]; thus, a biological response very close to that of alumina is expected from this material. In vitro tests with a cell type that is appropriate for the intended application (e.g., orbital fibroblasts, as suggested by Mawn et al. [43]) will be useful to complement the promising topographical results assessed in this work and to draw more definite conclusions about the suitability of these experimental implants.

## 4. Conclusions

Topographical investigations performed in this work revealed that all the considered materials for orbital implants had bumpy surface features, which should provide a benefit in terms of reduced abrasion of the delicate orbital tissues (especially conjunctiva) in contact with them. The only material that appeared spiky was commercial PMMA, but this spikiness was directed downward (negative skewness) and is not effective for abrasion. Glass-ceramic implants A and B exhibited highly favorable surface features as enucleation materials, similar to porous PE and alumina that are the today’s preferred devices by surgeons. Another potential advantage of these glass-ceramics is the more accessible cost compared to porous crystalline ceramics (hydroxyapatite and alumina implants), as glasses require lower processing temperatures associated with lower production expenses. 

## Figures and Tables

**Table 1:** Gross description of all the investigated materials, namely both the experimental implant materials GCA and GCB and the commercial implants selected as a reference (the two classes are separated by a continuous line). The major characteristics of implant materials (presence of crystalline phases, macropore size) come from product datasheet (for commercial implants) or previous assessment (for GCA and GCB [18]). PE: polyethylene; PMMA: poly(methyl methacrylate).

Implant Material	Specimen Shape And Size	Crystalline Phases	Total Porosity (vol %)	Mean Macropore Size (µm)
PMMA	ball, ~12.6 mm diameter	none	0	-
Silicone	ball, ~15.9 mm diameter	none	0	-
PE	ball, ~14.9 mm diameter	none	50	350
Alumina	ball, ~13.9 mm diameter	Al_2_O_3_	>75	500
GCA	cuboid, ~1 cm side	CaSiO_3_ (wollastonite)	~53	230
GCB	cuboid, ~1 cm side	Na_2_Ca_2_Si_3_O_9_ (combeite), Na_2_Ca_4_(PO_4_)_2_SiO_4_ (silicorhenanite), Ca_2_Mg(Si_2_O_7_) (akermanite)	~60	520

**Table 2:** Numerical values obtained by the topographical analysis; these data are represented in graphical form in Figure 4.

Material	S_q_ (nm)	S_a_ (nm)	S_k_	K_u_
PMMA	49 ± 21	36 ± 15	−1.1 ± 0.4	5.1 ± 1.3
Silicone	22 ± 4	17 ± 2	0.1 ± 0.5	1.3 ± 2.0
PE	411 ± 118	327 ± 95	−0.2 ± 1.0	0.4 ± 0.7
Alumina	287 ± 131	224 ± 104	−0.2 ± 0.4	0.5 ± 0.5
GCA	494 ± 304	466 ± 370	0.1 ± 0.4	1.2 ± 1.4
GCB	699 ± 270	556 ± 210	−0.3 ± 0.3	0.5 ± 1.0
